# Validation of disease-specific biomarkers for the early detection of bronchopulmonary dysplasia

**DOI:** 10.1038/s41390-022-02093-w

**Published:** 2022-05-20

**Authors:** Alida S. D. Kindt, Kai M. Förster, Suzan C. M. Cochius-den Otter, Andreas W. Flemmer, Stefanie M. Hauck, Andrew Flatley, Juliette Kamphuis, Stefan Karrasch, Jürgen Behr, Axel Franz, Christoph Härtel, Jan Krumsiek, Dick Tibboel, Anne Hilgendorff

**Affiliations:** 1 Institute of Computational Biology, Helmholtz Zentrum München, Munich, Germany; 2 Department of Analytical Biosciences, Leiden Academic Centre for Drug Research, Leiden University, Leiden, the Netherlands; 3 Member of the German Lung Research Center (DZL), Department of Neonatology, Perinatal Center, Dr. von Hauner Children’s Hospital, Ludwig-Maximilians University, LMU Hospital, Munich, Germany; 4 Member of the German Lung Research Center (DZL), Institute for Lung Biology and Disease and Comprehensive Pneumology Center with the CPC-M bioArchive, bioArchive, Helmholtz Zentrum München, Munich, Germany; 5 Center for Comprehensive Developmental Care (CDeCLMU), Ludwig-Maximilians University, LMU Hospital, Munich, Germany; 6 Intensive Care and Department of Pediatric Surgery, Erasmus Medical Center–Sophia Children’s Hospital Rotterdam, Rotterdam, the Netherlands; 7 Research Unit Protein Science, Helmholtz Zentrum München, Munich, Germany; 8 Monoclonal Antibody Core Facility, Helmholtz Zentrum München, Neuherberg, Germany; 9 Institute of Epidemiology, Helmholtz Zentrum München, Munich, Germany; 10 Member of the German Lung Research Center (DZL), Department of Internal Medicine V, Ludwig-Maximilians University, LMU Hospital, Munich, Germany; 11 Department for Pediatrics, University of Tübingen, Tübingen, Germany; 12 Department for Pediatrics, University of Schleswig-Holstein, Campus Luebeck, Germany; 13 Department for Pediatrics, University of Würzburg, Würzburg, Germany; 14 Institute for Computational Biomedicine, Department of Physiology and Biophysics, Weill Cornell Medicine, New York, NY USA; 15 Institute for Lung Biology and Disease and Comprehensive Pneumology Center with the CPC-M bioArchive, Helmholtz Zentrum München, Munich, Germany

## Abstract

**Objective:**

To demonstrate and validate the improvement of current risk stratification for bronchopulmonary dysplasia (BPD) early after birth by plasma protein markers (sialic acid-binding Ig-like lectin 14 (SIGLEC-14), basal cell adhesion molecule (BCAM), angiopoietin-like 3 protein (ANGPTL-3)) in extremely premature infants.

**Methods and results:**

Proteome screening in first-week-of-life plasma samples of *n* = 52 preterm infants <32 weeks gestational age (GA) on two proteomic platforms (SomaLogic^®^, Olink-Proteomics^®^) confirmed three biomarkers with significant predictive power: BCAM, SIGLEC-14, and ANGPTL-3. We demonstrate high sensitivity (0.92) and specificity (0.86) under consideration of GA, show the proteins’ critical contribution to the predictive power of known clinical risk factors, e.g., birth weight and GA, and predicted the duration of mechanical ventilation, oxygen supplementation, as well as neonatal intensive care stay. We confirmed significant predictive power for BPD cases when switching to a clinically applicable method (enzyme-linked immunosorbent assay) in an independent sample set (*n* = 25, *p* < 0.001) and demonstrated disease specificity in different cohorts of neonatal and adult lung disease.

**Conclusion:**

While successfully addressing typical challenges of clinical biomarker studies, we demonstrated the potential of BCAM, SIGLEC-14, and ANGPTL-3 to inform future clinical decision making in the preterm infant at risk for BPD.

**Trial registration:**

Deutsches Register Klinische Studien (DRKS) No. 00004600; https://www.drks.de.

**Impact:**

- The urgent need for biomarkers that enable early decision making and personalized monitoring strategies in preterm infants with BPD is challenged by targeted marker analyses, cohort size, and disease heterogeneity.
- We demonstrate the potential of the plasma proteins BCAM, SIGLEC-14, and ANGPTL-3 to identify infants with BPD early after birth while improving the predictive power of clinical variables, confirming the robustness toward proteome assays and proving disease specificity.
- Our comprehensive analysis enables a phase-III clinical trial that allows full implementation of the biomarkers into clinical routine to enable early risk stratification in preterms with BPD.

## Introduction

Risk stratification for preterm infants with chronic lung disease (CLD), i.e., bronchopulmonary dysplasia (BPD), early after birth is urgently needed to inform postnatal clinical decision making. As of now, physicians have to rely on the diagnosis at 36 weeks gestational age (GA), solely based on clinical criteria.^1^ Previous approaches aiming at the identification of such biomarkers have been largely limited by the predominant use of targeted marker analysis, non-sensitive detection techniques, and standard data analysis approaches as opposed to statistical modeling including clinical variables.^2,3^ In order to overcome these limitations, we identified a combination of three plasma markers (basal cell adhesion molecule (BCAM), sialic acid-binding Ig-like lectin 14 (SIGLEC-14), angiopoietin-like 3 protein (ANGPTL-3)) using unbiased proteome screening (SOMAscan^®^ assay, SomaLogic^®^, Boulder, CO),^4,5^ whose expression levels in the first week of life and after 28 days were significantly associated with later BPD development,^6^ complemented by their verification in paraformaldehyde tissue sections from autopsy lungs of infants with BPD.

In order to now evaluate the potential of these proteins to serve as biomarkers in clinical routine as early as in the first week of life and thereby improve current risk stratification for BPD, we designed a study approach validating the biomarkers’ expression in a larger patient cohort as well as an independent sample set while rigorously assessing different, clinically relevant performance criteria. These included the evaluation of the biomarkers’ added value for disease detection in comparison to the sole use of clinical risk factors, the transfer of the measurement technique to a clinically applicable assay as well as the assessment of the biomarkers’ disease specificity by the use of neonatal and adult cohorts suffering from CLD of different origin. The comprehensive analysis was designed to enable a phase II clinical trial for implementation of the identified biomarkers into routine care for preterm infants.

## Patients and methods

### Patient characteristics

Sample sets analyzed comprise a training (preterm training cohort) and a validation (preterm validation cohort) cohort of preterm infants, as well as additional samples from a small group of preterm infants recruited at a different study site. Disease specificity was addressed in a cohort of neonatal CLD (CLD-CDH cohort) and a cohort of adult CLD patients (adult CLD cohort).

#### Preterm training cohort

We analyzed a total of 55 plasma samples obtained in the first week of life (median day of life 4, range: 0–7) from preterm infants born <32 weeks GA (total number of patients: *n* = 55, median GA 27.2 weeks, range: 23.2–30.6; 45.5% males). All infants were born at the Perinatal Center in Munich and prospectively enrolled into the AIRR study (Attention to Infants @ Respiratory Risks) after written informed parental consent. The approval was assigned by the Ethics Committee of the Medical Faculty of Ludwig-Maximilians University in Munich (Ethical vote #195-07). The study was registered at the German Registry for Clinical Trials (No. 00004600; https://www.drks.de). Preterm infants were prospectively included following the in- and exclusion criteria published previously:^6^ inclusion of preterm infants born <32 weeks GA with the exception of severe congenital malformations (e.g., hypoplastic left-heart syndrome, severe hypoplasia of the lungs or congenital diaphragmatic hernia (CHD)), chromosomal abnormalities (e.g., trisomy 13 or 18), inborn errors of metabolism, and decision for palliative therapy directly after birth). Clinical variables were comprehensively monitored from birth to discharge (Table 1) using the following consented definitions: intrauterine growth restriction: birth weight below the 10th percentile; diagnosis and severity of respiratory distress syndrome (RDS): assessment of anterior-posterior chest radiographs according to Couchard et al.;^7^ chorioamnionitis: inflammatory alterations of the chorionic plate (histologic examination) or signs of maternal and fetal signs of infection;^8^ presence of early postnatal systemic infections (early-onset infection (eoi)): one or more clinical and laboratory signs of infection according to Sherman et al.^9^

**Table 1:** Patient characteristics cohort of preterm infants (Munich).

	Training cohort	Validation cohort
*n*	52	25
GA (weeks)	27.2 (23.2–30.6)	27.0 (23.6–31.0)
Birth weight (g)	798 (510–1590)	820 (480–1620)
IUGR	6 (11.5)	5 (20)
Gender (female/male)	29/23	12/13
ANCS	46 (88.5%)	21 (84%)
Chorioamnionitis	27 (51.9%)	10 (40%)
Early-onset infection (EOI)	14 (26.9%)	6 (24%)
RDS ≥3	11 (21.2%)	8 (32%)
Mechanical ventilation (days)	45 (0–109)	48 (6–129)
Oxygen supplementation (days)	32 (0–186)	44 (0–129)
Postnatal steroids	22 (42.3%)	12 (48%)
ROP	15 (28.9%)	5 (20%)
IVH	6 (11.5)	1 (4%)
NICU stay (days)	63 (9–113)	65 (9–150)
Bronchopulmonary dysplasia (BPD)		
None	24 (46.2%)	10 (40%)
Mild	15 (28.9%)	5 (20%)
Moderate	5 (9.6%)	2 (8%)
Severe	8 (15.4%)	8 (32%)
Pulmonary hypertension (PH) (≥2/3 syst. pressure)	0 (0%)	0 (0%)

Samples of the preterm training cohort were analyzed on two analysis platforms (SOMAscan^®^ assay (*n* = 33); Proximity Extension Assay (*n* = 19)) and validated in an independent sample set (preterm validation cohort) by enzyme-linked immunosorbent assay (ELISA, *n* = 25). Data are given as median and range or number and percent of total in group’s respective range. NICU stay not available in *n* = 1 infant in the preterm training cohort.

*GA* gestational age, *ANCS* antenatal corticosteroids, *RDS* respiratory distress syndrome, *ROP* retinopathy of prematurity, *IVH* intraventricular hemorrhage, *ICU* intensive care unit, *BPD* bronchopulmonary dysplasia.

BPD was defined according to the NICHD/NHLBI/ORD workshop^1^ based on the need for oxygen supplementation (>FiO_2_ 0.21) for at least 28 days, followed by a final assessment at 36 weeks postmenstrual age (PMA) or at discharge, whichever came first in preterm infants born <32 weeks GA. Disease grading accordingly assigned infants to having mild BPD (requirement of supplemental oxygen for 28 days, no need for oxygen supplementation at 36 weeks PMA) or moderate BPD (oxygen supplementation <FiO_2_ 0.30 at 36 weeks PMA), and severe BPD (oxygen supplementation >FiO_2_ 0.30 at 36 weeks PMA and/or positive pressure ventilation/continuous positive pressure) with each treatment referring to its continuous application and oxygen supplementation >12 h equaling one day of treatment.^1^ The infants’ oxygen saturation was assessed by standardized pulse oximetry. No infant was discharged from hospital before 36 weeks’ gestation.

#### Preterm validation cohort

To validate the results obtained, we analyzed expression levels for all three proteins in an independent sample set of the AIRR study collected in the first week of life (median day of life 0, range 0–5 days) by the use of commercially available enzyme-linked immunosorbent assay (ELISA). Preterm infants were included following the same in- and exclusion criteria as outlined above (total number of patients: *n* = 25, median GA 27.0 weeks, range 23.6–31.0; 52% males). In this cohort, *n* = 10 infants did not develop BPD (no BPD (40%)), *n* = 5 infants developed mild BPD (20%), and a total of 10 infants were diagnosed with either moderate BPD (*n* = 2 (8%)) or severe BPD (*n* = 8 (32%)) with no infant being discharged before 36 weeks’ gestation (Table 1).

In an additional step, a small group of preterm infants was recruited after written informed parental consent at a different study site following the same in- and exclusion criteria in order to mimic sampling conditions of a clinical trial, i.e., ongoing recruitment of small sample sets with random distribution of clinical characteristics; total number of patients: *n* = 8, median GA: 25.6 weeks, range: 24.1–29.0; median birth weight: 852 g, range: 520–1470 g; eoi *n* = 2 (25%), 75% males, *n* = 4 no BPD, *n* = 4 moderate/severe BPD). The approval was assigned by the Ethics Committee of the University of Schleswig-Holstein (Ethical vote #AZ 15-304).

#### Neonatal CLD-CDH cohort

To assess disease specificity of the biomarkers investigated, we repeated their measurement in a neonatal cohort of infants suffering from CDH with and without CLD. CLD was defined according to the need for mechanical ventilation and/or oxygen supplementation beyond day 28 of life, thereby following the BPD definition from the NICHD/NHLBI/ORD workshop for infants >32 weeks PMA.^1^ The infants were part of the VICI-trial^10^ and prospectively included after informed parental consent at the ErasmusMC Sophia Children’s Hospital in Rotterdam. The approval was assigned by the Ethics Committee of the University of Rotterdam, the Netherlands (Ethical vote #MEC-2006-260). The cohort included 21 neonates in total with a median GA 38.0 weeks (range 33.6–41.3), 33.3% males. Six infants did not develop CLD (no CLD-CDH) and nine infants developed CLD (CLD-CDH (survivors)). Six infants deceased (CLD-CDH (deceased)) (Table 2).

**Table 2:** Patient characteristics Neonatal CLD-CDH cohort (Rotterdam).

*n*		21
GA (weeks)		38.0 (33.6–41.3)
Gender (female/male)		(14/7)
Death		6 (28.6%)
Early-onset infection (EOI)	No	17 (81%)
	Yes	4 (19%)
Mechanical ventilation	No	8 (38.1%)
	Yes	10 (47.6%)
	NA	3 (14.3%)
O_2_ (day 28)	No	6 (28.6%)
	Yes	10 (47.6%)
	NA	2 (9.5%)
Severity CLD	No CLD	6 (28.6%)
	Mild CLD	8 (38.1%)
	Severe CLD	1 (4.8%)
PH (first echocardiogram)	No	6 (28.6%)
	PH (from 2/3 of syst. pressure on)	14 (66.7%)
	NA	1 (4.8%)
ECMO	No	13 (61.9%)
	Yes	8 (38.1%)
iNO	No	11 (52.4%)
	Yes	10 (47.6%)

Data are given as median and range or number and percent of total in group’s respective range.

*CLD* chronic lung disease, *ECMO* extra corporal membrane oxygenation, *EOI* early-onset infection, *GA* gestational age, *iNO* inhalative nitric oxide, *NA* not available, *PH* pulmonary hypertension.

#### Adult CLD cohort

Disease specificity of the biomarkers was furthermore assessed in a cohort of adult CLD patients after informed consent (CPC-M bioArchive, Munich, Ethics Committee of the Medical Faculty of Ludwig-Maximilians University in Munich (Ethical vote #19-629)) comprising samples from patients with idiopathic pulmonary fibrosis (IPF, total number of patients *n* = 21, median age 56 years (range 30–73), 76.7% males), chronic obstructive pulmonary disease (COPD, total number of patients: *n* = 26, median age 50 years (range 14–74), 58% males), and subjects free of lung disease according to clinical history from the KORA cohort^11^ (total number of patients: *n* = 25, median age 60 years (range 53–67), 52% males). KORA (Cooperative Health Research in the Region Augsburg) is a regional research platform for population-based surveys and subsequent follow-up studies with a focus on diabetes, cardiovascular, and lung diseases, including the impact of environmental factors.

### Sample analysis

#### Sampling processing

Serial whole blood samples (200 µl minimum each) obtained during routine laboratory blood drawings were collected using ethylenediaminetetraacetic acid neonatal collection tubes. After pseudonymization samples were processed for proteomic screening by centrifugation (1000 g, 5 min) before supernatants were aliquoted and stored at −80 °C. Time from sample collection to sample processing was standardized according to the study protocol (DRKS No. 00004600).

#### Proteomic analysis

Samples from the preterm training cohort were analyzed on two analysis platforms in three subsequent batches (SOMAscan^Ⓡ^ assays: 1st batch *n* = 16, 2nd batch *n* = 20; Proximity Extension Assay (PEA): *n* = 19; collected at day 4 of life (median), range: 0–7) followed by validation in a sample set of independent patients (preterm validation cohort, *n* = 25) that used a clinically applicable analysis technique, i.e., ELISA. Samples from an additional group of preterm infants (*n* = 8) were analyzed by PEA. Analysis for disease specificity comprised samples from cohorts with neonatal and adult CLD of different origin, i.e., *n* = 21 neonates in the CLD-CDH cohort (PEA (Olink-Proteomics^®^), *n* = 21) and *n* = 72 in the adult CLD cohort (SOMAscan^®^ assay (SomaLogic^®^)).

The SOMAscan^Ⓡ^ assay (SomaLogic^®^, Boulder, CO) uses 1129 individual high-affinity molecules (SOMAmer^®^—slow off-rate modified DNA aptamer—reagents) quantified on a custom Agilent hybridization array.^5,12^ The PEA (Olink-Proteomics^®^, Uppsala, Sweden) employs a matched pair of antibodies linked to unique oligonucleotides detected in multiplexed fashion in a high throughput fluidic chip system measuring 630 unique proteins.^13^ Both techniques are designed for the accurate quantification of human plasma proteins present in concentrations below picogram per milliliter using even low-amount samples. For PEA measurements, SIGLEC5/14 was detected in the identical aliquot by ELISA (R&D Systems, MN).

We validated the results obtained by the use of a clinically applicable method (ELISA) in an independent sample set obtained from *n* = 25 infants (preterm validation cohort, Table 1). The commercially available ELISAs targeted all three proteins and were performed according to the manufacturer’s instructions. Samples were measured in duplicates and diluted 1:100 for the SIGLEC5/14 ELISA (R&D Systems #DY1072), 1:10 for the BCAM ELISA (Thermo Scientific #EHBCAM), and 1:200 for the ANGPTL-3 ELISA (Ray Biotech #ELH-ANGPTL3). Readouts were obtained in a TECAN Spark ELISA reader (Tecan Trading AG, Switzerland).

### Statistical analysis

Three outliers were detected by principal component analysis (prcomp function, R framework, log_2_-transformed, and pareto scaled data) and removed from further analysis and summary statistics. Preterm training cohort: protein expression obtained from two different sample sets analyzed by SOMAscan^®^ (SomaLogic^®^) (1st batch *n* = 16, 2nd batch *n* = 17) and one sample set analyzed by PEA (Olink-Proteomics^®^) (*n* = 19) were batch corrected using the combat function from the sva package (version 3.36) in R (version 4.0; Supplementary Fig. 1). Next, validation was performed in an independent cohort of 25 preterm infants, in which protein concentrations were determined by ELISA. Further, we independently analyzed results obtained by PEA in samples from eight preterm infants recruited at a different study site. Analysis for disease specificity comprised samples from cohorts with neonatal and adult CLD of different origin, i.e., *n* = 21 neonates in the CLD-CDH cohort (PEA (Olink-Proteomics^®^), *n* = 21) and *n* = 72 in the adult CLD cohort (SOMAscan^®^ assay (SomaLogic^®^)). Area under the curve (AUCs) were calculated using a leave-one-out cross-validation with a generalized linear model that compared no BPD infants to (a) only moderate and severe BPD cases or (b) all BPD cases as binary outcome and log_2_ transformed, pareto scaled protein expression data together with the clinical variables GA, birth weight, eoi, and sex as covariates, which are the most important risk factors associated with BPD development.^14^ These variables were analyzed with and without protein expression levels as covariates in a total of 32 different combinations including a null model. The model with the highest AUC and lowest Akaike’s Information Criterion (AIC) was the one used to perform the prediction of BPD and the continuous variables of duration of mechanical ventilation [days], oxygen supplementation [days], and neonatal intensive care unit (NICU) duration [days]. All prediction models for the preterm training cohort were calculated using a leave-1-out cross-validation with a linear regression model using the covariates from the best model comprising protein expression levels and GA. The models applied for the CLD-CDH cohort and adult CLD cohort, i.e., IPF and COPD, were corrected for GA (neonates) or age (adult patients). Three tests (Bartlett test, Fligner–Killeen test, and Levene test (from the car package)) were used to check for equal variances before ANOVA testing. All protein concentrations were log_2_ transformed and pareto scaled prior to statistical analysis.

**Fig. 1:**
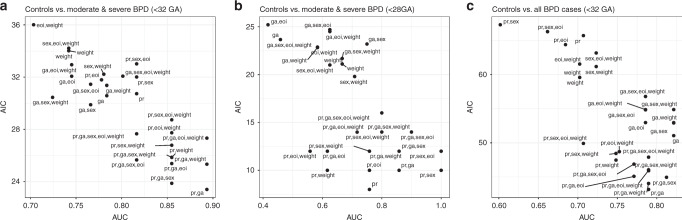
Improved performance of BPD prediction by novel plasma protein biomarkers detected in the first week of life. Protein markers (SIGLEC-14, BCAM, ANGPTL-3) significantly improve performance of BPD prediction models solely based on clinical variables. AIC vs. AUC for 31 of the 32 analyzed models (excluding the null model from the graphs) including protein expression levels (pr), gestational age (GA), sex, birth weight (weight) and early-onset infection (eoi) for **a** no BPD vs. moderate and severe BPD (<32 weeks GA), for **b** no BPD vs. moderate and severe BPD (<28 weeks GA), and for **c** no BPD vs. all BPD Grades (<32 weeks GA). AIC Akaike’s Information Criterion, AUC area under the curve, BPD bronchopulmonary dysplasia, BPD grades: 0 = no BPD, 1 = mild BPD, 2 = moderate BPD, 3 = severe BPD.

## Results

In summary, the final dataset after exclusion of outliers comprised 52 samples in the preterm training cohort (*n* = 24 no BPD (46.2%), *n* = 15 mild BPD (28.9%), *n* = 5 moderate BPD (9.6%) and *n* = 8 severe BPD (15.4%)) and 25 samples in the preterm validation cohort (Table 1). In addition, 8 samples from a different study site were analyzed to mimic a clinical study setting. To determine disease specificity, 21 samples from a neonatal CLD-CDH cohort and 72 samples from an adult CLD cohort were analyzed.

### Protein markers significantly improve the performance of BPD prediction models based on clinical variables

We compared 32 models that included protein expression levels for BCAM, SIGLEC-14, and ANGPTL-3 as well as critical risk factors for BPD development, i.e., GA, sex, birth weight, and eoi, which are the most important risk factors associated with BPD development,^14^ to determine the model with the highest sensitivity and specificity (highest AUC) for the separation of moderate and severe BPD cases from no BPD while explaining the data only by the most important variables (lowest AIC). We show that the combination of the three protein markers together with GA (BPD~BCAM+SIGLEC-14+ANGPTL-3+GA) best predicted BPD in the first week of life with an optimized AUC (0.87) and AIC (23.40), thereby being superior to the other models tested, e.g., “GA alone” (AUC = 0.87, AIC = 30.46), and the null model (AIC = 49.97; Fig. 1a). The model furthermore demonstrated high sensitivity (0.92), specificity (0.86), accuracy (0.89) and positive predictive value (0.80) as well as test accuracy (F_1_-scores: 0.89). These results were confirmed when restricting the analysis to very immature infants, i.e., <28 weeks GA, again demonstrating superiority with increased AUC (0.86) and decreased AIC (10.0) when compared to other models tested (null model AIC: 24.91; Fig. 1b).

When including all BPD grades, the model comprising the protein markers together with GA demonstrated improved performance (AIC: 43.16) when compared to the model with GA alone (51.0) and the null model (73.77; Fig. 1c). In contrast, AUC levels are comparable between the models when comparing no BPD vs. all BPD cases (protein levels and GA: AUC = 0.83, GA alone: AUC = 0.84; Fig. 1c).

### Protein markers enable BPD prediction for all disease grades with significant accuracy at birth

**Fig. 2:**
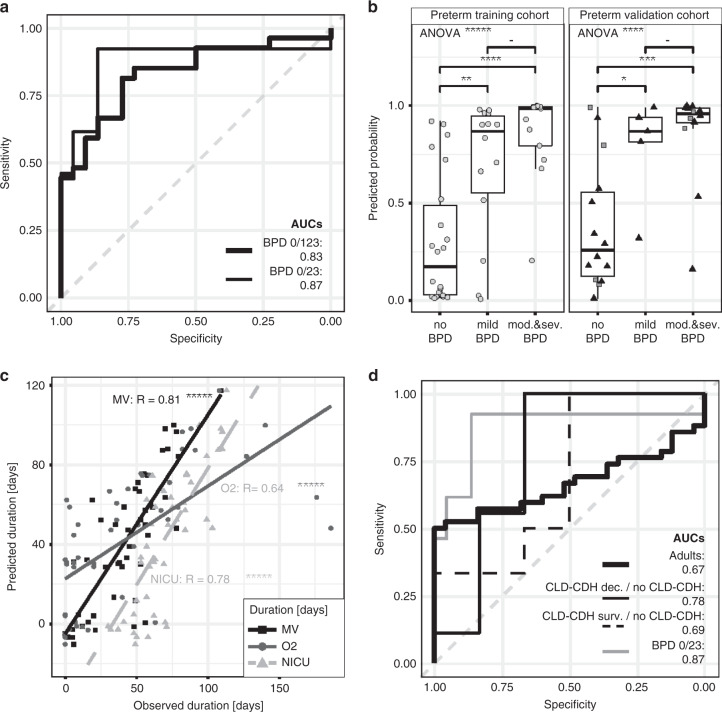
Disease specific biomarkers predict BPD severity with significant accuracy. Protein markers enable BPD prediction for all disease grades with significant accuracy at birth while demonstrating disease specificity. **a** AUC values calculated for protein expression levels and GA: black bold line (preterm training cohort): no BPD vs. all BPD cases, AUC = 0.83; black line (preterm training cohort): no BPD vs. moderate and severe BPD AUC = 0.87. **b** Predicted probability of no BPD vs. all BPD cases resulting from leave-1-out cross-validation using the model with protein expression levels and GA for preterm training cohort (ANOVA *p* = 1.4 × 10^−5^, gray filled circles); *t*-test *p* values for: no BPD vs. mild BPD *p* = 3.4 × 10^−3^; no BPD vs. (mod./severe) BPD, *p* = 1.4 × 10^−6^; mild BPD vs. (mod./severe) BPD, *p* = 0.13; and the ELISA measurements (black triangles) for the preterm validation cohort (ANOVA *p* = 2.4 × 10^−4^); *t*-test *p* values for: no BPD vs. mild BPD *p* = 0.024; no BPD vs. (mod./severe) BPD, *p* = 1.4 × 10^−4^; mild BPD vs. (mod./severe) BPD, *p* = 0.56. Samples from another study site (*n* = 8, Luebeck, gray filled squares) show homogeneous distribution within the preterm validation cohort. **c** Prediction analysis (leave-1-out model, preterm training cohort) for mechanical ventilation [days] (black squares, black solid line; *r* = 0.81, *p* = 2.9 × 10^−12^), O_2_ supplementation [days] (gray solid line, gray circles; *r* = 0.64, *p* = 7.2 × 10^−7^) and duration of NICU stay [days] (gray dashed line, gray triangles; *r* = 0.78, *p* = 7.1 × 10^−11^). **d** Specificity for BPD prediction in the preterm in comparison to neonatal and adult CLD patients: bold black line (adults): AUC = 0.67; black line (CLD-CDH cohort): CLD-CDH deceased vs. no CLD-CDH infants AUC = 0.78; dashed line (CLD-CDH cohort): CLD-CDH survivors vs. no CLD-CDH infants, AUC = 0.69; gray line (preterm training cohort: no BPD vs. moderate and severe BPD (AUC = 0.87) as reference.

For the model comprising plasma protein levels and GA, receiver operating characteristic curves for no BPD vs. mild, moderate, and severe BPD and no BPD vs. moderate and severe BPD indicate high sensitivity for BPD prediction in the first week of life (Fig. 2a). The model successfully separates infants according to their risk for later BPD while considering different disease grades: no BPD vs. mild, moderate and severe BPD, ANOVA *p* value = 1.4 × 10^−5^ (leave-1-out cross-validation); no BPD vs. mild BPD (*t*-test *p* value = 3.4 × 10^−3^); no BPD vs. moderate and severe BPD (*t*-test *p* value = 1.4 × 10^−6^; Fig. 2b (left panel)). The results obtained were confirmed by ELISA measurements in an independent sample set (preterm validation cohort); ANOVA *p* value = 2.4 × 10^−4^; *t*-test *p* values no BPD vs. mild BPD *p* = 0.024; no BPD vs. moderate/severe BPD, *p* = 1.4 × 10^−4^; Fig. 2b (right panel)). In an additional analysis, we demonstrated the successful separation according to the risk for BPD development at birth by the proteins in combination with GA in a small sample set recruited at a different study site (*n* = 8; Fig. 2b (right panel, gray filled squares)).

The model furthermore correctly predicts the need for mechanical ventilation (duration in days; observed vs. predicted: *r* = 0.81, *p* value = 2.9 × 10^−12^, MAE = 8.60, RMSE = 10.61), and oxygen supplementation (duration in days; observed vs. predicted: *r* = 0.64, *p* value = 7.2 × 10^−7^, MAE = 23.20, RMSE = 32.12) as well as the duration of NICU stay [(duration in days; observed vs. predicted: *r* = 0.78, *p* value = 7.1 × 10^−11^, MAE = 9.57, RMSE = 12.70; Fig. 2c) in the preterm training cohort.

### Protein markers show specificity for BPD prediction in the preterm when compared to neonatal and adult CLD patients

Application of the model including the protein levels and GA in a cohort of neonatal patients with CLD due to CDH (CLD-CDH cohort) showed no discrimination between neonates with CDH and age-matched infants with CLD-CDH when analyzing survivors (AUC 0.69; Fig. 2d). The protein markers only allowed the separation of maximum disease, i.e., fatal outcome from all survivors including no CLD-CDH and CLD-CDH patients (AUC 0.78; Fig. 2d). In adult patients with CLD (adult CLD cohort), protein levels did not separate cases with COPD or IPF from pulmonary healthy controls with sufficient power (Fig. 2d).

The study showed very good power to separate BPD phenotypes in the preterm training cohort (no BPD vs. all BPD grades: 87%; no BPD vs. moderate/severe BPD: 97%) and good power to separate cases in the CLD-CDH cohort (no CLD-CDH vs. CLD-CDH (survivors): 60%; CLD-CDH (fatal outcome) vs. no CLD-CDH: 72%).

## Discussion

BPD is a multifactorial disease and remains the most serious lung condition in neonates born premature due to its significant mortality and morbidity. Despite the clinical significance, the diagnostic process solely relies on clinical criteria assessed at 36 weeks PMA. The relatively late diagnosis only inadequately addresses both the need for early risk stratification as well as the diseases’ multifaceted pathology that includes the rarefication of the gas exchange area, interstitial remodeling, and airway pathology, now clustered at a late stage in a non-discriminatory diagnosis.^14^ The diagnostic gap with regard to timeliness and accuracy is reflected by the limitations of clinical trials aiming at the implementation of new therapeutic strategies^15–19^ and underscores the need for new markers enabling today’s clinicians to early and sensitively identify CLD in the preterm infant.

We therefore followed a rigorous approach to evaluate protein markers with significant potential to serve as future biomarkers for BPD prediction as early as in the first week of life, previously identified by us using unbiased proteome screening.^6^ Based on these findings, we first applied a generalized linear model to identify the best combination of proteins and clinical risk factors for BPD prediction and demonstrated the significant impact of the protein expression levels on improving BPD prediction when compared to clinical markers only. Second, we addressed the markers’ ability to successfully predict BPD grades as well as the duration of mechanical ventilation, oxygen supplementation, and intensive care treatment. Third, we successfully validated the biomarker results in an independent cohort of preterm infants using a clinically applicable measurement technique, i.e., ELISA and in a final step defined disease specificity in neonatal and adult patients suffering from CLD of different origins.

With the assessment of critical factors that can affect the biomarkers’ clinical performance including their validation in different cohorts and their resilience toward different measurement techniques, we overcame significant limitations of previous studies.

The assessment of 32 models identified the combination of GA together with the protein expression levels as the best model for BPD prediction while demonstrating the reproducible impact of the proteins on disease stratification when compared to known clinical covariates. The comparison between infants with higher disease grades to infants that did not develop BPD unequivocally identifies infants at risk, whereas the comparison of all BPD cases to patients without BPD is limited by the clinical heterogeneity of cases with mild BPD.^14^ Nonetheless, the model demonstrates good sensitivity and specificity to identify all BPD cases. Confirmation of the results in infants <28 weeks GA further supports the clinical value of the model by demonstrating superiority in a subcohort of very immature infants, in which GA alone is assumed to predict BPD development with significant power.^20,21^

The demonstration of the model’s potential to separate infants with different BPD grades from infants without the disease in the first week of life, and the validation of the results in an independent sample set with a clinically applicable protein measurement technique, i.e., ELISA, not only underscores the independence of the results from the measurement technique applied but adds significant insight into the performance of the potential biomarkers that is unmet by previous studies.

Reviewing and discussing the identification of reliable markers to predict BPD by previous studies, the use of clinical disease indicators including intrauterine growth restriction,^22^ low GA or birth weight, male sex,^23^ RDS, and invasive mechanical ventilation,^24^ sepsis, asphyxia, and chorioamnionitis,^25^ as well as race or ethnicity,^26,27^ and mode of delivery^28,29^ show only moderate predictive value.^21^ The limitations might be enhanced by significant changes in the diagnostic or therapeutic process applied to very low birth weight infants over time.^30,31^

Addressing the need for additional markers, a variety of studies aimed at identifying protein-based biomarkers, with the majority of studies centered around the detection of inflammation.^2,3^ Here, even the development of multivariate logistic regression models for the outcome of BPD or death at PMA of 36 weeks using protein expression levels of 25 cytokines as suggested by the Neonatal Research Network of the National Institute of Child Health and Human Development (NICHD NRN)^32^ did not improve disease prediction significantly, potentially due to the use of a pre-selected set of markers as opposed to their identification by unbiased screening. At the same time, markers derived from metabolomic analysis that showcased a cluster of 53 interesting metabolites associated with BPD development^33^ are most likely limited by the markers sensitivity toward sample collection and processing as well as the analytical platform used. Furthermore, marker detection in tracheal aspirates requires intratracheal intubation for sample acquisition, which becomes rarer with current postnatal treatment strategies.^34^ Despite significantly informing other fields of lung disease^35,36^ genetic BPD markers, although informing pathophysiologic understanding,^2,37,38^ have not been shown to significantly contribute to risk stratification until now.^39–42^ In contrast, the use of miRNAs already shows promising potential for BPD diagnosis and treatment.^3,43,44^

In conclusion, the use of protein-based biomarkers may thus be today’s method of choice when generated by unbiased screening such as in the study by Arjaans et al. that used the SOMAmer technology^®^ for the identification of biomarkers that enable the detection of vascular disease in the preterm infants.^45^ The approach succeeded showing early postnatal changes in circulating angiogenic peptides in association with disease, only limited by the lack of a validation cohort.

Likely, the reflection of the three most important processes of BPD pathophysiology by the three proteins supports their strength for early risk stratification: As the presence of the immune-activating SIGLEC-14^46,47^ has been previously associated with invasive infections in human newborns^48^ and the host response to viral airway infections,^49^ it holds promising potential to reflect the degree of pulmonary inflammation characterizing the BPD lung.^50–53^ The laminin receptor BCAM likely mirrors the process of tissue remodeling and its associated cellular cross-talk,^51,54^ thereby potentially reflecting BPD “activity”.^55^ In line with the findings of Arjaans et al.,^45^ ANGPTL-3 also is associated with angiogenic signaling, playing a role in endothelial development and survival, whereas BCAM holds functions in the basal membrane thereby reflecting significant changes to the surrounding niche of the developing alveoli.

Pulmonary expression of the three markers demonstrated in our previous study by the use of paraformaldehyde tissue sections from autopsy preterm BPD lungs^6^ supports their potential as indicators of BPD pathophysiology and demonstrates that the circulating proteins largely originate from lung tissue in contrast to other studies.^2,3,45^

In order to further add to previous studies and to showcase clinical applicability of the biomarker, we simulated the circumstances of a clinical trial by the use of a small dataset characterized by random differences in the patient’s characteristics and successfully demonstrated the fit of the data obtained in the distribution of the expression levels of the larger sample sets. Furthermore, we showed disease specificity of the biomarkers for BPD prediction by their application in a neonatal (CLD-CDH cohort) and adult CLD cohort (adult CLD cohort). Here, the proteins in combination with the confounder GA did not allow the stratification of CDH neonates that developed CLD but only separated infants with fatal outcome, whereas adult patients suffering from emphysema (COPD) or lung fibrosis (IPF) could not be separated from pulmonary healthy individuals at all when using the biomarker combination.

While successfully demonstrating the clinical value of the three biomarkers, limitations of our study include (i) cohort size, which we, however, consider adequate for a study in ELGAN’s (extremely low GA newborns) and (ii) the fact that despite the prospective study design, patients cannot be considered randomly assigned to disease groups. To partially compensate for these limitations, we underscored clinical relevance when demonstrating the proteins’ ability to predict main risk factors associated with BPD, i.e., need for mechanical ventilation, oxygen supplementation and NICU stay in the context of GA, as well as by their validation in an independent sample set, resulting in comparable results despite differences in cohort characteristics (Table 1). The limited cohort size was remedied by combining the expression profiles of the three proteins from several proteomic platforms and correcting the observed batch effect as well as the subsequent validation using a different measurement technique, i.e., ELISA in an independent sample set. Although the use of the SOMAscan^®^ or the PEA assay allows for the extensive study of a large number of peptides, peptides playing a role in the pathogenesis of BPD, however, might be missed. These candidates, when identified and validated by other studies, could be added to the model for further improvement.

In conclusion, we demonstrated the promising potential of the identified proteins to inform clinical decision making while considering critical clinical variables. The study significantly adds to previous biomarker studies in the field as it addresses disease specificity and the biomarkers’ robustness toward changes in cohort characteristics or measurement technique.^2,3,56^ The currently prepared clinical trial aims to prove the biomarkers’ benefit for guiding clinical care. Future studies will have to address the potential of the biomarkers to inform disease monitoring, supported by our previous findings demonstrating stable protein expression levels at day 28 of life^6^ or to identify disease subtypes dominated by inflammation, matrix remodeling, or vascular pathology.^57,58^

## Supplementary information

Supplementary table

Supplementary information figure

## Data Availability

The data are available on request by contacting the corresponding author.
