# Supplementary figures and images for: Validation of disease-specific biomarkers for the early detection of bronchopulmonary dysplasia
Source: Pediatr Res. 2022 May 20;93(3):625–32. doi: 10.1038/s41390-022-02093-w (PMC9988689; doi:10.1038/s41390-022-02093-w)

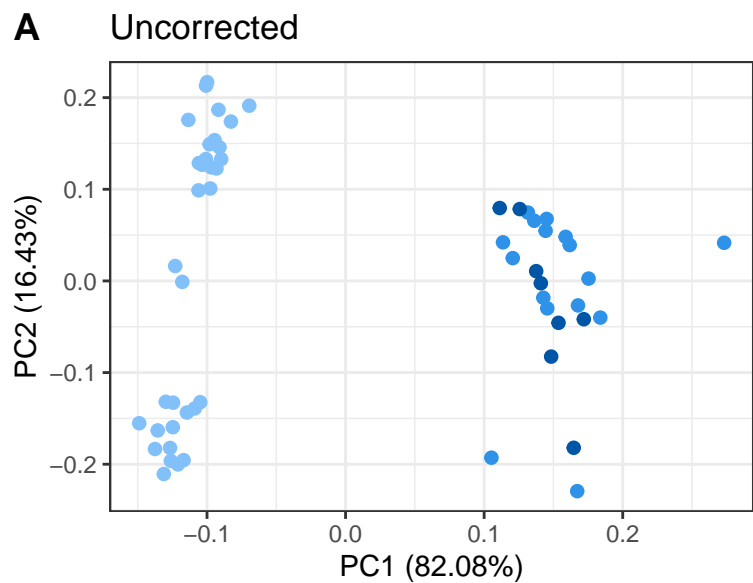

● Olink (Lubeck) ● Olink (Munich) ● SomaLogic (Munich)

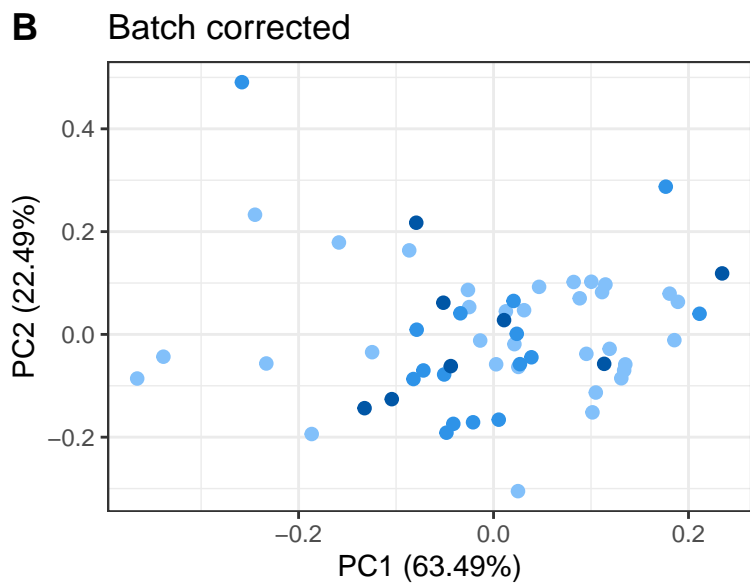

Supplement: Supplementary file 2 — Supplementary information figure [file 41390_2022_2093_MOESM2_ESM.pdf]
